# Combined targeting of HER-2 and HER-3 represents a promising therapeutic strategy in colorectal cancer

**DOI:** 10.1186/s12885-019-6051-0

**Published:** 2019-09-05

**Authors:** Lena-Christin Conradi, Melanie Spitzner, Anna-Lena Metzger, Merle Kisly, Peter Middel, Hanibal Bohnenberger, Jochen Gaedcke, Michael B. Ghadimi, Torsten Liersch, Joseph Rüschoff, Tim Beißbarth, Alexander König, Marian Grade

**Affiliations:** 1 Department of General, Visceral and Pediatric Surgery, University Medical Center Goettingen, Robert-Koch-Str. 40, 37075 Goettingen, Germany; 2 Department of Pathology, Pathologie Nordhessen, Kassel, Germany; 3 Department of Pathology, University Medical Center Goettingen, Goettingen, Germany; 4 Department of Medical Statistics, University Medical Center Goettingen, Goettingen, Germany; 5 Department of Gastroenterology and gastrointestinal Oncology, University Medical Center Goettingen, Goettingen, Germany

**Keywords:** Colorectal cancer, HER-2, HER-3, Targeted therapy, Inhibitors

## Abstract

**Background:**

Abrogation of growth factor-dependent signaling represents an effective therapeutic strategy for patients with colorectal cancer (CRC). Here we evaluated the effectiveness of targeting the epidermal growth factor (EGF) receptors HER-2 and HER-3 in the three cell lines LS513, LS1034 and SW837.

**Methods:**

Treatment with HER-2-specific antibodies trastuzumab and pertuzumab resulted in a mild reduction of cellular viability. In contrast, the antibody-drug conjugate T-DM1 mediated a strong and dose-dependent decrease of viability and Akt phosphorylation.

**Results:**

The most striking effects were observed with the dual tyrosine kinase inhibitor lapatinib, and the Pan-ErbB inhibitor afatinib. Selectively, the effect of EGF receptor inhibition was augmented by a combination with 5-fluorouracil and oxaliplatin. Finally, high expression of HER-3 was detected in 121 of 172 locally advanced rectal cancers (70.3%). In conclusion, inhibition of EGF receptors effectively blocks downstream signaling and significantly impairs viability of CRC cells. However, the effectiveness of receptor inhibition highly depends on the inhibitors’ mode of action, as targeting HER-2 alone is not sufficient.

**Conclusion:**

Since HER-2 and HER-3 are expressed in a relevant number of patients, targeting both receptors may represent a promising therapeutic strategy for CRC.

**Electronic supplementary material:**

The online version of this article (10.1186/s12885-019-6051-0) contains supplementary material, which is available to authorized users.

## Background

Colorectal cancer represents the third most common cancer and the second leading cause of cancer-related deaths in the United States and Western Europe [1, 2]. However, despite implementation of multimodal treatment approaches and novel targeted therapeutics within the last two decades [3, 4], the occurrence of distant metastases still limits the prognosis of affected patients. In this context, up to 50% of patients with CRC develop metastatic disease recurrence, predominantly in the liver and the lung, and, until now, surgical resection represents the only curative strategy [5–7]. Unfortunately, resectability is technically not always feasible, and disease recurrence after metastasis resection is frequently observed [8, 9]. Consequently, there is an urgent clinical need to develop novel agents and treatment strategies to inhibit metastatic cancer progression.

In metastatic CRC, treatment regimens were commonly based on 5-fluorouracil (5-FU) and, recently, in combination with irinotecan or oxaliplatin [10]. Due to the lack of specificity of these drugs, there have been major initiatives in targeted-therapy approaches. A primary focus was EGF receptor signaling, which plays a key role in CRC development and progression [11–13]. Major clinical trials, including recent data from the CELIM study, have demonstrated that initially unresectable CRC liver metastases can be surgically removed after combined EGFR inhibition and chemotherapy (CTx), resulting in a better survival of these patients [14, 15]. Unfortunately, most CRC develop resistance against EGFR-targeting agents, which ultimately limits this therapeutic strategy [16, 17]. Therefore, the evaluation of alternative therapeutic targets is crucial for the implementation of innovative treatment approaches. In this context, the transmembrane receptors HER-2 and HER-3 represent interesting candidates.

HER-2, a member of the EGF receptor family of receptor tyrosine kinases (Erb), commonly referred as ErbB2, represents a prognostic biomarker in breast cancer and has been a molecular target for many years [18, 19]. Recently, HER-2 inhibition has also been integrated into therapeutic strategies for metastatic gastric cancer [20, 21]. Among other studies, the ToGA-trial demonstrated HER-2 positivity in about 20–30% of adenocarcinomas of the stomach and gastro-esophageal junction [22], and a survival benefit upon treatment with trastuzumab using a specifically modified immunohistochemistry (IHC) scoring algorithm, which differed from breast cancer [20]. While data about the prognostic and functional relevance of HER-2 expression are still limited for most gastrointestinal malignancies [21, 23], we have recently reported HER-2 positivity in more than 20% of primary rectal cancer [24], and overexpression of HER-2 in nearly 10% of CRC-derived liver metastases [25]. Moreover, we observed overexpression of another member of the EGF receptor family, HER-3, in approximately 70% of CRC-derived liver metastases [25]. This observation is of high clinical interest because novel HER-3 inhibitors have been recently developed and are currently being tested within early phase clinical trials [26, 27].

In the present study, we determined the protein expression of HER-2 and HER-3 in 12 CRC cell lines using immunocytochemistry (ICC). Selected cell lines were treated with the HER-2-specific antibodies trastuzumab or pertuzumab, which either prevent ligand binding or dimerization of HER-2 with other HER receptors. Additionally, cells were incubated with the antibody-drug conjugate T-DM1, the dual tyrosine kinase inhibitor lapatinib, and the irreversible Pan-ErbB (HER-1/HER-2/HER-4) inhibitor afatinib. Specific targeting of Erb receptors was combined with 5-FU and oxaliplatin, which represents a standard regime in the clinical setting. Finally, we evaluated the frequency of HER-3 protein expression in patients with primary rectal cancer using IHC.

## Methods

### Cell lines and cell culture

Human CRC cell lines HT29, SW403, SW837, SW1116, LS513, LS1034, Caco-2, SW1463, SW480, SW620, HCT116, and LS411N were obtained from the American Type Culture Collection (ATCC, Manassas, VA) and cultured in their recommended media (Invitrogen, Karlsruhe, Germany), supplemented with 2 mM L-glutamine (Lonza, Verviers, Belgium) and 10% fetal bovine serum (Biochrome, Berlin, Germany). Periodically, mycoplasma contamination was excluded using the MycoAlert® Mycoplasma Detection Kit (Lonza, Cologne, Germany), and cell-line cross-contamination was excluded using short tandem repeat profiling [28]. Relevant characteristics of these cell lines are summarized in Table 1.

**Table 1:** Genetic characteristics and HER-2/HER-3 immunostaining of 12 CRC cell lines

Cell Line	*TP53* Mutation	*KRAS* Mutation	*APC* Mutation	MMR Status	*HER-2* Mutation	HER-2 ICC	HER-3 ICC
HT29	+	-	+	MSS	-	1+	3+
SW403	+	+	+	MSS	-	1+	1+
SW837	+	+	+	MSS	-	2+	1+
SW1116	+	+	+	MSS	-	2+	3+
LS513	-	+	-	MSS	-	2+	3+
LS1034	+	+	+	MSS	-	2+	3+
Caco-2	+	-	+	MSS	-	2+	1+
SW1463	+	+	+	MSS	-	1+	3+
SW480	+	+	+	MSS	+	0	0
SW620	+	+	+	MSS	-	0	0
HCT116	-	+	-	MSS	-	0	0
LS411N	+	-	+	MSS	+	2+	1+

*MMR* mismatch repair, *MSI* microsatellite-instable, *MSS* microsatellite-stable, *ICC* immunocytochemistry

### Drugs

Trastuzumab, pertuzumab, and T-DM1 (Roche, Penzberg, Germany) were obtained by the local pharmacy of the University Medical Center in Goettingen. Small-molecule inhibitors afatinib and lapatinib were purchased from Santa Cruz (Dallas, TX), and 5-FU and oxaliplatin from Sigma (Munich, Germany).

### Cellular viability assays

Cellular viability was determined using the CellTiter-Blue® reagent (Promega, Madison, WI), as previously described [29]. Briefly, cell lines growing in log-phase were seeded at different densities (8000 cells per well for LS513; 6000 cells for LS1034; and 6000 cells for SW837, respectively) into black clear bottom 96-well plates (Corning, Corning, NY). Cells were allowed to adhere overnight, and drugs were added with increasing concentrations. Twenty-four, 48, and 72 h upon treatment start, reduction of resazurin to resorufin was measured using a plate reader (VICTOR™ X4, Perkin Elmer, Waltham, MA) according to the manufacturer’s instructions. Cellular viability of antibody-treated cells was compared to untreated cells, and viability of inhibitor-treated cells was compared to DMSO-controls, as previously described [30]. All experiments were performed as three independent replicates, with three technical replicates per plate.

### Western blot analysis

Cell lines were seeded into six-well plates (10^6^ cells per well) with increasing concentrations for the indicated drugs. Twenty-four hours later, cells were stimulated with 100 ng/ml neuregulin (NRG, Cell Signaling, Danvers, MA) for 10 min at 37 °C. Subsequently, cells were lysed using RIPA buffer (50 mM Tris, 150 mM NaCl, 0.5% Na-deoxycholate, 1% NP-40, 2 mM EDTA) followed by sonification. Finally, 20 μg of whole-cell protein lysate was resolved on a 10% Bis-Tris gel (Roth, Karlsruhe, Germany) at 30 mA per gel. Proteins were transferred by wet blotting (Criterion™ blotter, Bio-Rad, Hercules, CA) onto a PVDF membrane (Merck-Millipore, Billerica, MA), and probed with primary antibodies p-Akt (1:1000; Cell Signaling), Akt (1:1000; Cell Signaling), and Actin (1:2000; Sigma, Saint Louis, MO) at 4 °C over night. On the next day, membranes were incubated for 2 h with the secondary antibody goat-anti-rabbit-HRP (1:30,000; Acris, Hiddenhausen, Germany). Signals were detected using ECL Luminata forte (Merck-Millipore) and a CCD camera system (LAS 4000mini; GE Healthcare, Munich, Germany).

### Immunostaining

Cell lines with 70 to 80% confluence were trypsinized, washed with PBS, and fixed with buffered 4% formaldehyde (AppliChem, Darmstadt, Germany) over night at room temperature. Subsequently, cells were incubated with increasing concentrations of ethanol (60–100%) for 30 min, followed by incubation with isopropanol and xylene for 30 min. Finally cells were covered with hot paraffin for 10 min and embedded into a paraffin block.

HER-2 immunostaining was conducted using a PATHWAY® anti-HER-2/neu (4B5) rabbit monoclonal antibody (Ventana Medical Systems, Mannheim, Germany) on a Ventana BenchMark XT immunostainer (Ventana, Tucson, AZ), visualized by the ultraView Universal DAB Detection Kit (Ventana Medical Systems), as previously described [24, 25]. HER-3 expression was determined in both cell lines and primary rectal cancer specimens using the anti c-erbB-3/HER-3 rabbit monoclonal antibody (clone SP71; Zytomed Systems, Berlin, Germany).

### HER-2 and HER-3 scoring

For HER-2 scoring, we used an established protocol, which has been developed within the ToGA-trial and which is now being used to determine HER-2 protein expression in patients with adenocarcinoma of the stomach and the gastroesophageal junction [20, 22, 31, 32]. Importantly, we have previously used this protocol to score HER-2 and HER-3 expression in primary rectal cancers and CRC liver metastases [24, 25].

Cell lines or cancer cells from formalin-fixed paraffin embedded patient samples were considered ICC 2+ if at least 10% of the tumor cells had a medium membrane staining for HER-2 or HER-3, respectively, at high magnification (10x, 20x magnified), or ICC 3+ if at least 10% of the tumor cells had a strong membrane staining at low magnification (2.5x, 5x magnified). No membrane staining was scored ICC 0, and weak membrane staining in at least 10% of the tumor cells was defined as ICC 1+ (40x magnified).

### Statistical analysis

Significant effects in cellular viability assays were analyzed using logistic regression with generalized linear models (glm) and analysis of variance (ANOVA). In the linear model, the cellular viability (in percent) was modeled as dependent on different replicates, different duration effects (24 h, 48 h, 72 h), a log10 dose effect and dose:duration interaction effects. Model comparisons were performed via ANOVA using the F-Test to assess whether the addition of the duration, dose or interaction variables adds significant information to the model. Statistical analyses were conducted using R statistical computing environment version 3.1.1. The (estimated) half maximal effective concentration (EC_50_) was estimated based on the fitted logistic regression curves for each measurement series. If the EC_50_ lies outside the range of measured doses, extrapolations can be inaccurate and lead to very large estimates. Comparisons of two measurement series were performed using a similar logistic regression model. Here, an additional drug combination effect plus all interaction effects were estimated. The ANOVA *P*-value for the combination effect indicates that the drug combination displays a significantly different effect from the treatment with one drug alone (Additional file 5: Table S1).

The association of HER-3 expression levels with other clinico-pathological parameters was assessed using Fisher’s exact test. Survival rates were supplied by means of Kaplan-Meier analysis and tested using the Cox proportional hazards model. Time to recurrence (TTR) was defined as the interval between surgical resection of the primary tumor and disease recurrence, and cancer-specific survival (CSS) as time from surgical resection to Colorectal cancer-related death. The *P*-value was set to *P* < 0.05 to be considered statistically significant. Survival analysis was performed using the R package survival.

## Results

### HER-2 and HER-3 status in CRC cell lines

Since Erb receptor positivity has been reported in a substantial proportion of colorectal cancer patients, we analyzed the expression of HER-2 and HER-3 in a large set of colorectal cancer cell lines. These cell lines were specifically chosen because we had extensively characterized them before [29, 33–35]. Using immunocytochemistry, strong expression of HER-3 was detected in five out of 12 cell lines (ICC score ≥ 2+; Table 1), whereas high (3+) or borderline (2+) expression of HER-2 was present in 50% of our model system (ICC score ≥ 2+; Table 1). This indicates that a relevant proportion of CRC cell lines overexpresses either HER-2 or HER-3 or a combination of both. Three cell lines with borderline HER-2 overexpression (defined as ICC score ≥ 2) and/or HER-3 overexpression were selected for further experimentation, i.e., LS513, LS1034, and SW837 (Additional file 1: Figure S1; Table 1, highlighted in red).

### Influence of HER-2 inhibition on cellular viability

**Fig. 1:**
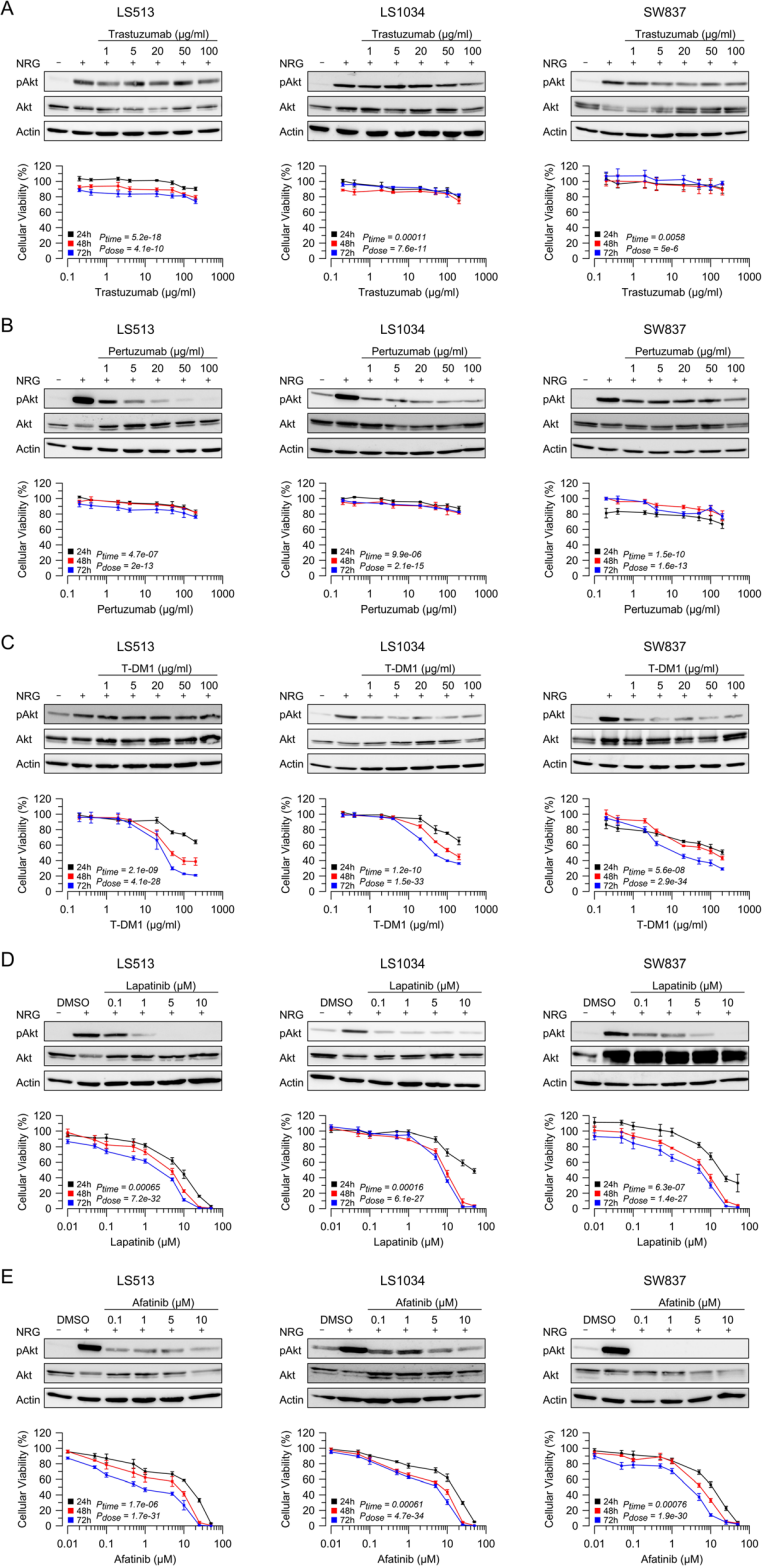
Influence of HER-2/HER-3 inhibition on cellular viability. Cellular viability of LS513, LS1034, and SW837 cells was determined 24 h (black curve), 48 h (red curve), and 72 h (blue curve) after treatment with increasing concentrations of trastuzumab (**a**), pertuzumab (**b**), T-DM1 (**c**), lapatinib (**d**), and afatinib (**e**). To assess the inhibitory effect on downstream signaling, cells were treated with increasing concentrations of the respective inhibitors for 24 h, stimulated with 100 ng/ml neuregulin for 10 min, and referred to Western blot analysis. All experiments were performed in triplicate, independently repeated three times. The respective *P*-values and the (estimated) EC_50_ for all drugs and time points are listed in Additional file 5: Table S1

To functionally characterize the impact of HER-2 expression on cell survival, HER-2 positive cell lines were treated with increasing doses of the HER-2-specific antibodies trastuzumab and pertuzumab, which either prevent ligand binding or dimerization of HER-2 with other HER receptors, with the antibody-drug conjugate T-DM1, the dual tyrosine kinase inhibitor lapatinib, and the irreversible Pan-ErbB (HER-1/HER-2/HER-4) inhibitor afatinib. As shown in Fig. 1, treatment with trastuzumab or pertuzumab resulted in a rather mild reduction of cellular viability in all cell lines (Fig. 1a, b). For pertuzumab (Fig. 1b), but not for trastuzumab (Fig. 1a), this observation was accompanied by decreased Akt phosphorylation at Serine 473, indicating reduced Akt activity. In contrast, T-DM1 mediated a strong and dose-dependent decrease of cellular viability in all cell lines, accompanied by a distinct reduction of Akt phosphorylation (Fig. 1c). The most striking effect, however, was observed after treatment with lapatinib, which inhibits the EGFR and HER-2 receptor (Fig. 1d), or afatinib, which irreversibly alters HER-1, HER-2,, and HER-4 signaling (Fig. 1e). Treatment with either lapatinib or afatinib resulted in a complete abrogation of cellular viability for prolonged time periods. The respective *P*-values and the (estimated) half maximal effective concentrations (EC_50_) for all drugs and time points are listed in Additional file 5: Table S1.

These findings suggest that HER-2 inhibition results in reduced cellular viability of CRC cells, but the effect is dependent on the mode of action of the respective inhibitor. It also indicates that blockade of HER-2 alone is not sufficient for appropriate cell growth inhibition.

### Effectiveness of dual HER-2 inhibition on cellular viability

**Fig. 2:**
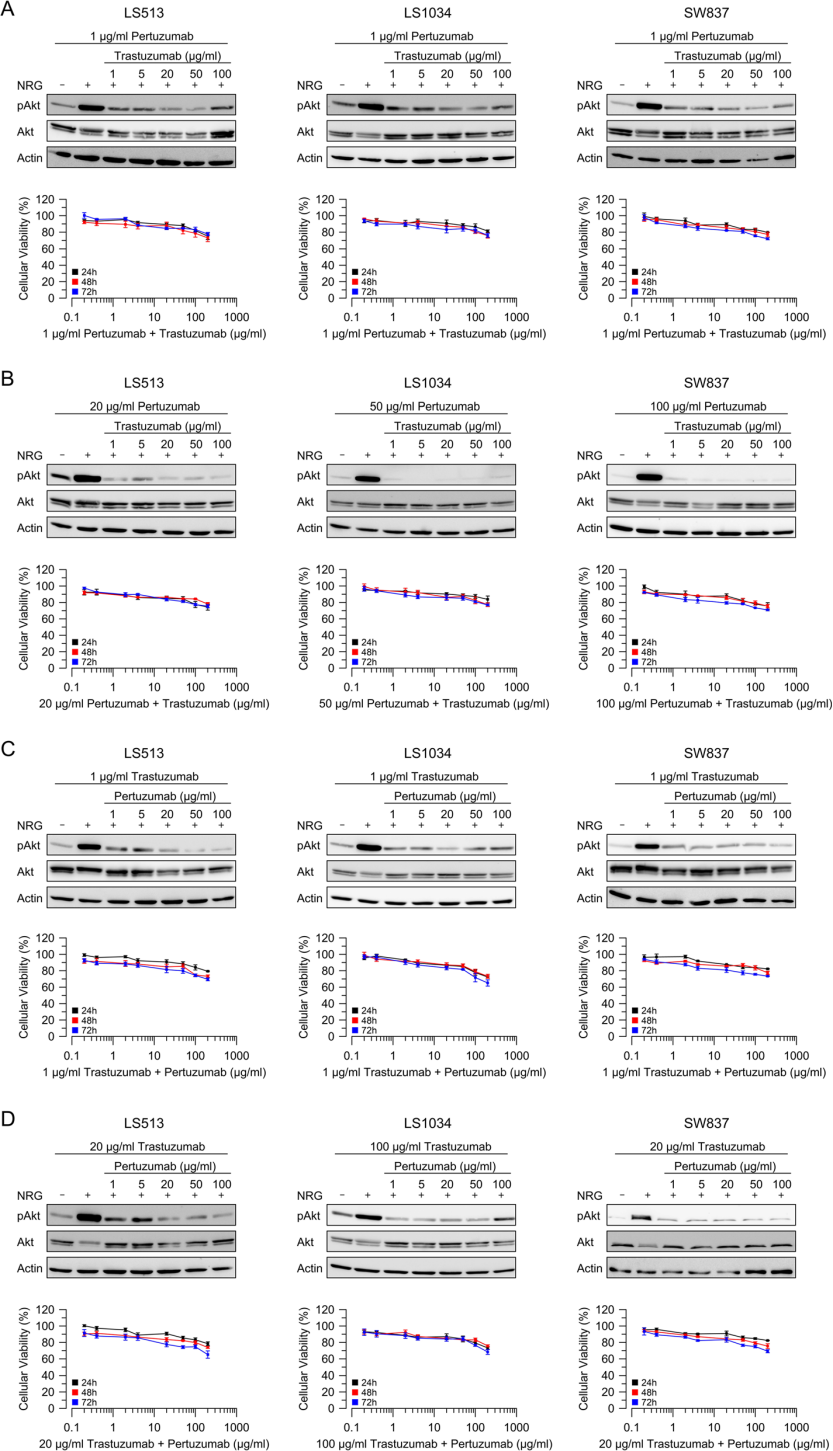
Cellular viability upon combined targeting of HER-2/HER-3. Cellular viability of LS513, LS1034, and SW837 cells was assessed 24 h (black curve), 48 h (red curve), and 72 h (blue curve) after treatment with various combinations of trastuzumab and pertuzumab. The inhibitory effect on downstream signaling was evaluated by Western blot analysis. All experiments were performed in triplicate, independently repeated three times. (**a** + **b**) Two different doses of pertuzumab were combined with increasing concentrations of trastuzumab. (**c** + **d**) Two different doses of trastuzumab were combined with increasing concentrations of pertuzumab. The respective *P*-values and the (estimated) EC_50_ for all drugs and time points are listed in Additional file 5: Table S1

Because neither treatment with trastuzumab nor pertuzumab markedly decreased cellular viability, we tested the combination of both drugs, which is routinely used for breast cancer patients [36], and which is currently being tested for gastric cancer patients in the INNOVATION trial (ClinicalTrials.gov Identifier: NCT02205047). Two different doses of pertuzumab were combined with increasing concentrations of trastuzumab, and vice versa (Fig. 2). Despite striking inhibitory effects on downstream signaling, as demonstrated by decreased Akt phosphorylation, the combination of these drugs had only mild effects on cellular viability, regardless of the duration of the treatment (Fig. 2, Additional file 5: Table S1). These results corroborate the interpretation that specific inhibition of HER-2 is neither effective nor sufficient for significant alteration of cellular viability in colorectal cancer cells.

### Effectiveness of HER-2 inhibition combined with 5-FU and oxaliplatin

Since systemic treatment of metastatic CRC patients frequently involves a combination of targeted therapy with either 5-FU and/or oxaliplatin [37], we now aimed to evaluate the effectiveness of combining HER-2 inhibition with 5-FU and oxaliplatin. To define a treatment base line we first determined the impact of 5-FU, and oxaliplatin, alone on cellular viability [34], and tested distinct combinations thereof (Additional file 2: Figure. S2 and Additional file 3: Figure S3, Additional file 5: Table S1). For further combination experiments with Erb inhibitors, we selected 5-FU and oxaliplatin concentrations that decreased relative cellular viability to approximately 60–80% (Additional file 3: Figure S3).

**Fig. 3:**
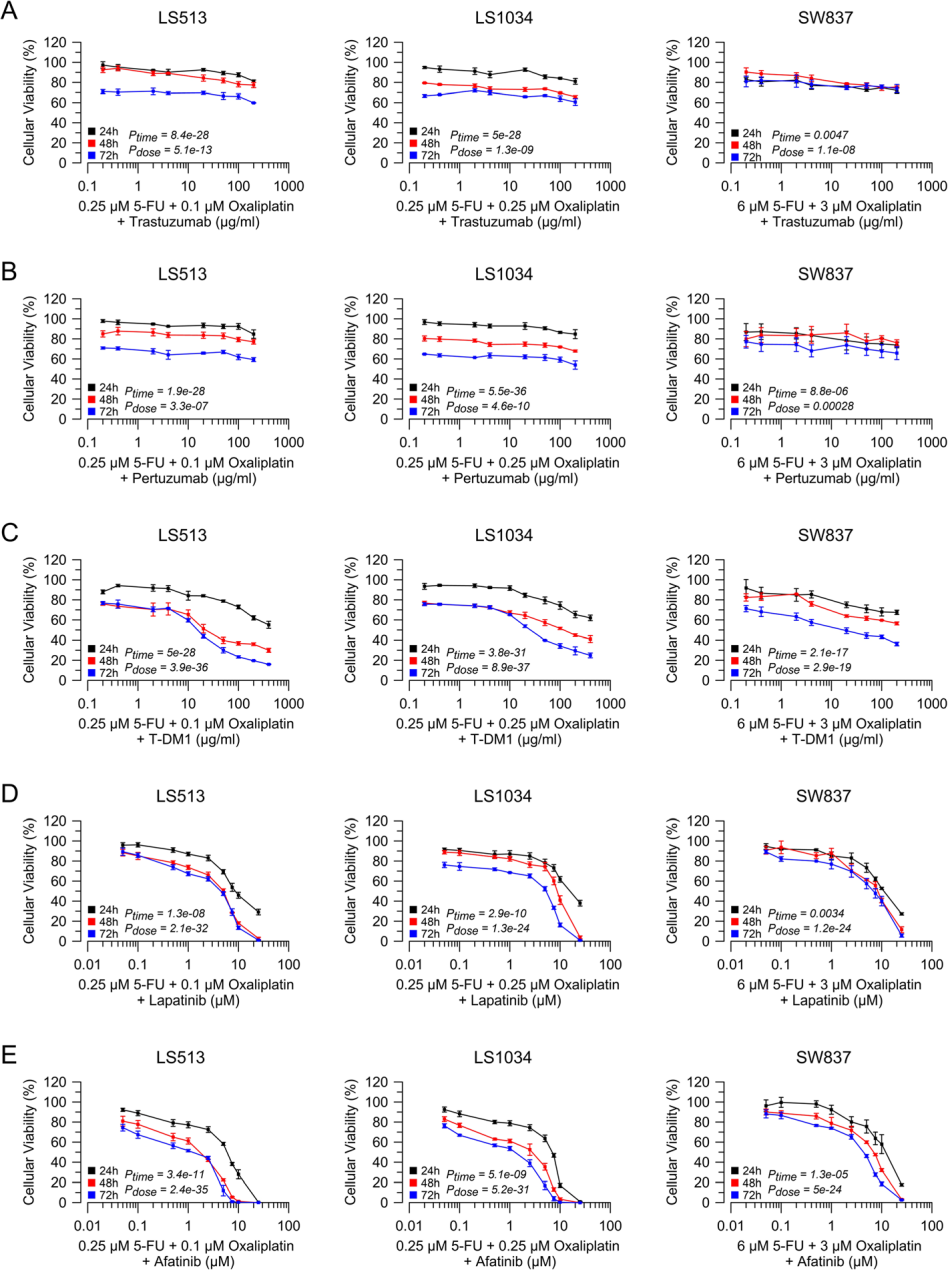
Effectiveness of HER-2/HER-3 inhibition combined with 5-FU and oxaliplatin. Cellular viability of LS513, LS1034, and SW837 cells was assessed 24 h (black curve), 48 h (red curve), and 72 h (blue curve) after treatment with 5-FU and oxaliplatin combined with trastuzumab (**a**), pertuzumab (**b**), T-DM1 (**c**), lapatinib (**d**), and afatinib (**e**). All experiments were performed in triplicate, independently repeated three times. The respective *P*-values and the (estimated) EC_50_ for all drugs and time points are listed in Additional file 5: Table S1

Trastuzumab combined with 5-FU and oxaliplatin exhibited a relatively moderate effect, which was time-dependent, but dose-independent, in LS513 and LS1034 cells (Fig. 3a). A similar result was observed for pertuzumab and 5-FU/oxaliplatin (Fig. 3b). In contrast, T-DM1 combined with 5-FU/oxaliplatin mediated a strong dose- and time-dependent effect on cellular viability (Fig. 3c). The strongest effect, however, was detected upon treatment with either lapatinib (Fig. 3d) or afatinib (Fig. 3e) in combination with 5-FU and oxaliplatin. In both cases, the observed result was dose- and time-dependent.

**Fig. 4:**
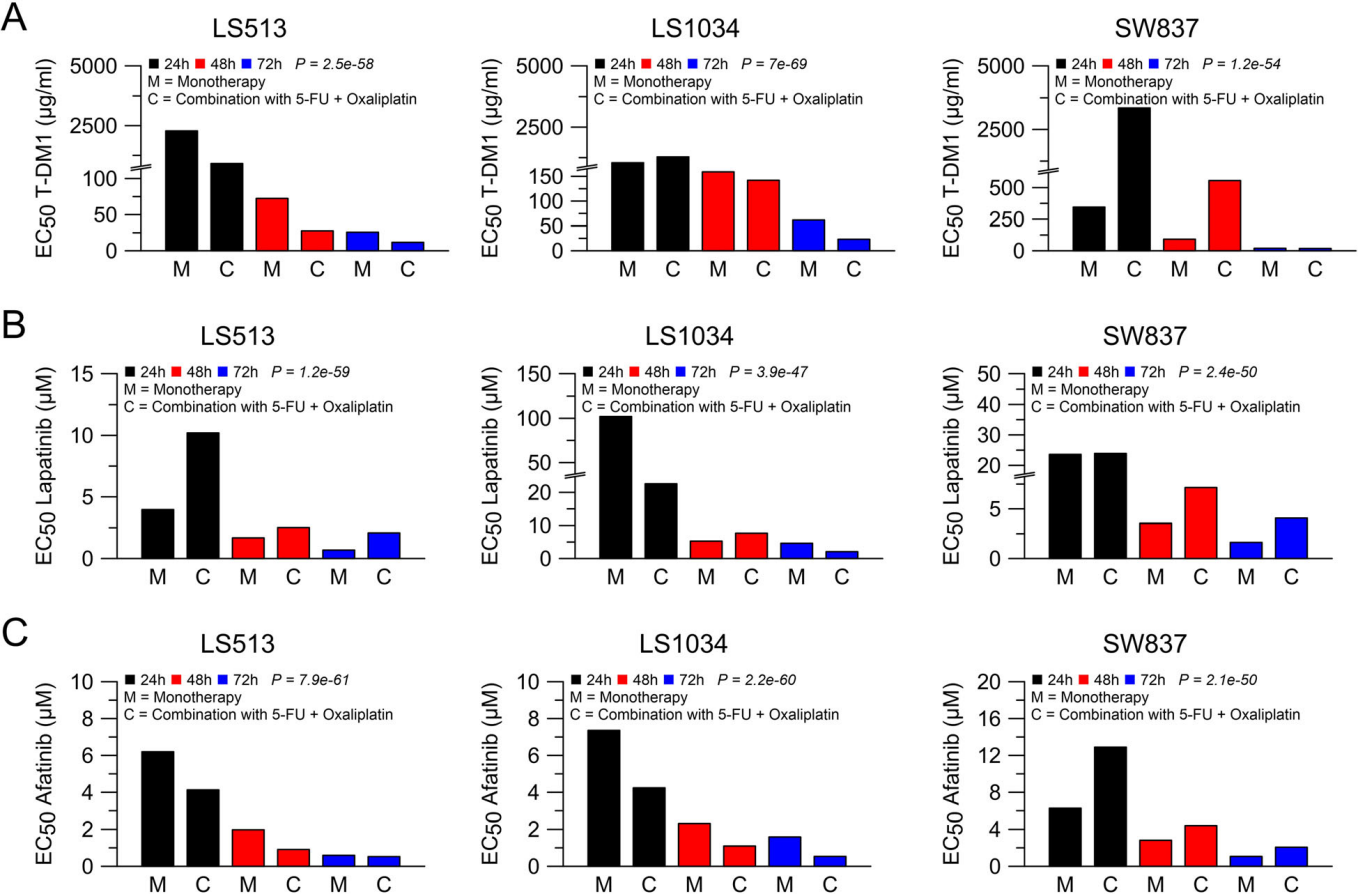
Comparison of anti-HER-2/HER-3 monotherapy and a combination with 5-FU and oxaliplatin. Displayed are the respective EC_50_ 24 h (black bar charts), 48 h (red bar charts), and 72 h (blue bar charts) after treatment. Compared to monotherapy, the combination of T-DM1 (**a**), lapatinib (**b**), and afatinib (**c**) with 5-FU and oxaliplatin mediated stronger effects only in LS513 and LS1034, but not in SW837. Not shown are the bar charts for trastuzumab and pertuzumab, as they were not time- and dose-dependent

Finally, we aimed to explore synergistic effects of Erb inhibition and conventional chemotherapy in colorectal cancer cells. Therefore, we statistically compared the effect of Erb inhibition (Fig. 1) with combined treatment of Erb inhibitors and 5-FU/oxaliplatin (Fig. 3). We applied a statistical model that fitted logistic regression curves, and used ANOVA analysis to determine significant differences between these treatments. Interestingly, the combination of 5-FU/oxaliplatin with T-DM1 (Fig. 4a), lapatinib (Fig. 4b), and afatinib (Fig. 4c) mediated stronger effects on cellular viability in LS513 and LS1034 colorectal cancer cells.

### HER-3 protein expression in primary rectal cancer

**Fig. 5:**
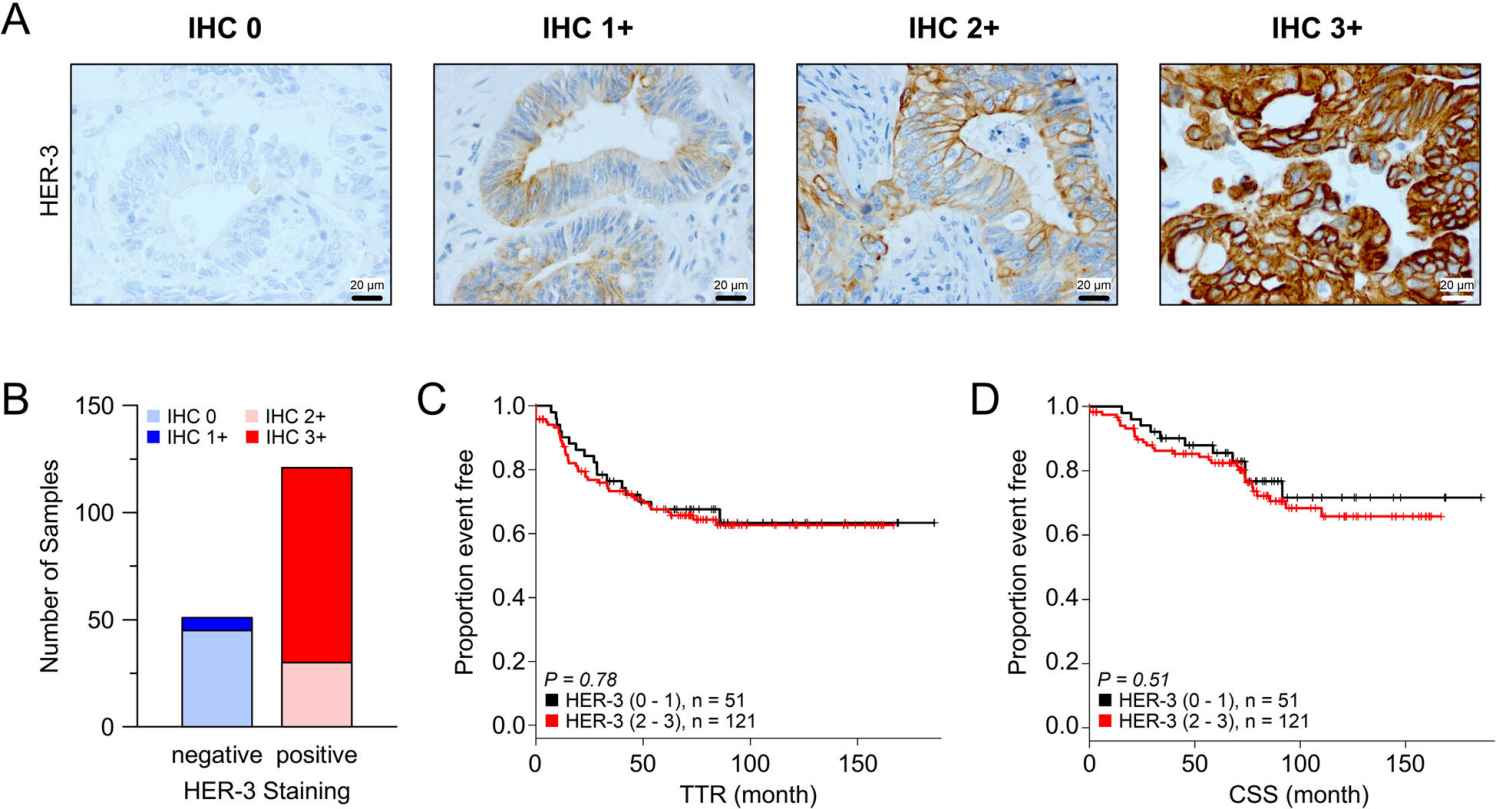
HER-3 protein expression of primary rectal cancer visualized by immunohistochemistry staining. **a** shows different intensities of HER-3 expression and the grading from no staining (IHC 0) to an intensive staining for HER-3 (IHC 3+). The positivity rate for HER-3 protein expression and the distribution of different staining grades within the analysed cohort of 172 CRC patients are depicted in the bar graph in **b**. Kaplan-Meier curves showing the time to recurrence (TTR) (**c**) and the cancer specific overall survival (**d**) in patients with tumors negative or with low expression of HER-3 (IHC 0–1+) versus a high HER-3 expression (IHC 2–3+)

As a result of these experiments, inhibition of the HER-2 receptor alone is not sufficient to abrogate colorectal cancer cell growth. Simultaneous inhibition of additional members of the Erb receptor family altered cellular viability and downstream signaling more effectively. Both findings suggest that unspecific, i.e., simultaneous targeting of the Erb receptor family is a more promising approach in colorectal cancer treatment. Obviously, a pivotal requirement for this hypothesis is a relevant expression of these Erb receptors in CRC patients. Therefore, we assessed the expression of HER-3 in 172 tissue specimens of locally advanced rectal cancer samples using immunohistochemistry (Fig. 5a). We found high expression (IHC 2+ and IHC 3+) in more than 70% of our tumor samples (*n* = 121, Fig. 5b). Heterogeneity or focal HER-3 expression was detected in 52.3% of the tissues. Importantly, HER-3 expression significantly correlated with HER-2 expression (*P* = 0.01, Additional file 4: Figure S4). There was no correlation of HER-3 expression levels and clinico-pathological findings such as UICC stage (*P* = 0.7) or tumor regression grading upon neoadjuvant chemoradiation (*P* = 0.61). With a mean follow-up time of 78.2 months, patients with high HER-3 expression showed a comparable time to recurrence (TTR, *P* = 0.78) and cancer specific survival (CSS, *P* = 0.51) as compared to patients with low HER-3 expression (Fig. 5c, d).

Collectively, our data indicate that a relevant proportion of CRC cell lines and primary rectal cancer express HER-2 and HER-3. Importantly, inhibition of these receptors effectively blocks intracellular signaling and significantly impairs the viability of CRC cells in vitro. However, the effectiveness of receptor inhibition highly depends on the inhibitors’ mode of action, and combined inhibition of EGF receptor family members seems to be more effective than individual targeting of HER-2.

## Discussion

Given its high incidence in the Western world, treatment of CRC remains an important interdisciplinary task. Although innovative surgical concepts and the implementation of multimodal treatment strategies have considerably improved both local control and oncological outcome [38–40], systemic treatment of CRC patients with distant metastases remains a major clinical challenge. In this context, advances were obtained by combining cytostatic drugs such as 5-FU, oxaliplatin or irinotecan, and by the discovery and successful targeting of key signaling pathways, which promote colorectal carcinogenesis. Prime examples are the pharmacological inhibition of the vascular endothelial growth factor (VEGF) or the epidermal growth factor receptor (EGFR) in selected patients, which has become clinical routine [37, 41].

However, and despite initial responses to these therapeutic approaches, secondary resistance frequently evolves over time, ultimately resulting in treatment failure [16, 42]. The underlying mechanisms that lead to treatment resistance are quite complex and heterogeneous [43]. Recent work demonstrated that prolonged inhibition of the EGF receptor (EGFR/ErbB1) leads to selection of Ras mutations as well as an increased expression of other members of the ErbB family, which can replace EGFR in EGF-mediated oncogenic signaling [44, 45]. The two most prominent members of the ErbB family, which may substitute ErbB1 to escape EGFR inhibition, are HER-2 (ErbB2) and HER-3 (ErbB3). Especially the role of HER-2 has been described as a keystone in EGF-mediated growth activation in breast or gastric cancer [19, 46]. Based on our present results and on previous analyses of primary rectal adenocarcinomas and CRC liver metastases, we found both HER-2 and HER-3 overexpressed in a substantial proportion of CRC [24, 25], and in CRC cell lines. In addition, activating HER-2 mutations have been identified in CRC patients within the TCGA project as well as in several CRC cell lines [11, 47]. More recently, activating HER-2 mutations were also detected in Lynch-like CRC [48]. Consequently, both receptors represent attractive therapeutic targets.

Despite its activity in breast or gastric cancer [19, 46], monotherapy with HER-2 inhibitors trastuzumab or pertuzumab only slightly reduced the viability of HER-2 positive CRC cells. Moreover, and in contrast to previous results in breast cancer [49], the favorable effect of dual inhibition of HER-2 by simultaneous application of both antibodies showed only mediocre activity on CRC cell lines. A potential explanation why both antibodies, which specifically target the HER-2 receptor from outside the tumor cell, lack activity could be an intact intracellular tyrosine kinase activity resulting from heterodimerization with other ErbB family members or a constitutively active tyrosine kinase activity. Congruent with this assumption, inhibition of the tyrosine kinase activity of the HER-2 receptor by either lapatinib or afatinib dramatically impaired cellular viability in vitro. This effect was even more pronounced when treatment was combined with 5-FU and/or oxaliplatin, reflecting the clinically more relevant situation. Of note, the HERACLES phase-II trial recently tested as a proof of concept a combination of trastuzumab and lapatinib in patients with HER-2 positive metastatic CRC that were primarily resistant to cetuximab or panitumumab [50]. This study demonstrated that approximately 5% of K-RAS exon 2 wild-type metastatic CRC are HER-2 positive, which is comparable to other malignancies with druggable molecular targets. Importantly, the treatment was well tolerated, and about 1/3 of the patients experienced either partial or complete response [50].

The strongest impairment of cellular viability in our analyses, however, was observed upon treatment with the Pan-ErbB inhibitor afatinib, suggesting that other members of the ErbB family may be involved in EGF-mediated oncogenic signaling in colorectal cancer cells. These results together with the finding that HER-3 is expressed in a substantial proportion of CRC patients and CRC cell lines highlight the clinical rationale to simultaneously target members of the ErbB receptor family. Importantly, antibodies targeting HER-3 such as MM-121 (ClinicalTrials.gov: NCT01451632), RG7116 (ClinicalTrials.gov: NCT01482377) and U3–1287 are currently being tested in several clinical trials across various patient populations, including CRC patients. In cancers with ligand-dependent activation of HER-3, several studies suggest therapeutic potential of anti-HER-3 substances [51]. Recently, HER-3 was identified as predictive factor for clinical outcome in K-RAS wild-type CRC patients treated with cetuximab [52]. An ongoing clinical study evaluates treatment with MM-121 plus cetuximab versus MM-121 in combination with cetuximab plus irinotecan in CRC (ClinicalTrials.gov: NCT01451632). Another multicenter study is recruiting participants to evaluate RG7116 alone, RG7116 in combination with cetuximab, or RG7116 plus erlotinib in patients with metastatic and/or locally advanced HER-3 positive solid tumors (ClinicalTrials.gov: NCT01482377).

## Conclusion

In conclusion, selective inhibition of the HER-2 receptor alone does not seem to represent a promising therapeutic strategy for CRC treatment, in contrast to breast cancer or other cancers of the gastro-intestinal tract. In clear contrast, simultaneous inhibition of different members of the ErbB receptor family dramatically abrogated cellular viability of CRC cells in vitro. Since both HER-2 and HER-3 are overexpressed in a relevant proportion of primary CRC and CRC liver metastases, targeting of HER-2 and HER-3 simultaneously may be considered as a potential therapeutic strategy in these patients upon failure of EGFR inhibition.

## Additional files

Additional file 1: **Figure S1.** HER-2 and HER-3 status of CRC cell lines. Twelve CRC cell lines were analyzed for membrane expression of HER-2 and HER-3. Overexpression of HER-3 (ICC ≥ 2+) was detected in five cell lines, whereas HER-2 was overexpressed (ICC ≥ 2+) in six cell lines. Depicted are representative HER-2 and HER-3 stainings of LS513, LS1034, and SW837 cells, paraffin embedded. (PNG 5582 kb)

Additional file 2: **Figure S2.** Dose-response curves for oxaliplatin. Cellular viability of LS513 (A), LS1034 (B), and SW837 (C) cells was determined 24 h (black curve), 48 h (red curve), and 72 h (blue curve) after treatment with increasing concentrations of oxaliplatin. All experiments were performed in triplicate, independently repeated three times. (PNG 247 kb)

Additional file 3: **Figure S3.** Effect of a combination of 5-FU and oxaliplatin. Cellular viability of LS513, LS1034, and SW837 cells was determined 24 h (black curve), 48 h (red curve), and 72 h (blue curve) after treatment with various concentrations of oxaliplatin and 5-FU. (A + B) Different doses of oxaliplatin were combined with increasing concentrations of 5-FU. (C + D) Different doses of 5-FU were combined with increasing concentrations of oxaliplatin. All experiments were performed in triplicate, independently repeated three times. (PNG 964 kb)

Additional file 4: **Figure S4.** Correlation of HER-2 and HER-3 protein expression in the 127 rectal cancer resection specimens as determined by immunohistochemical staining for HER-2 and HER-3 respectively. Immunohistochemical scoring was performed in 3 different grades (no expression = 0, weak = 1 intermediate = 2 and strong = 3. (PNG 167 kb)

Additional file 5: **Table S1.** EC50s of LS513, LS1034, SW837. (DOC 125 kb)

## Data Availability

The datasets used and/or analysed during the current study are available from the corresponding author on reasonable request.

## Abbreviations

CRC: Colorectal cancer
CSS: Cancer specific survival
CTx: Chemotherapy
EC_50_: Half maximal effective concentrations
EGFR: Epidermal growth factor receptor
Erb: EGF receptor family of receptor tyrosine kinases
GLM: Generalized linear models
HER: Human epidermal growth factor receptor
ICC: Immunocytochemistry
IHC: Immunohistochemistry
