# Supplementary figures and images for: Combined targeting of HER-2 and HER-3 represents a promising therapeutic strategy in colorectal cancer
Source: BMC Cancer. 2019 Sep 5;19:880. doi: 10.1186/s12885-019-6051-0 (PMC6727342; doi:10.1186/s12885-019-6051-0)

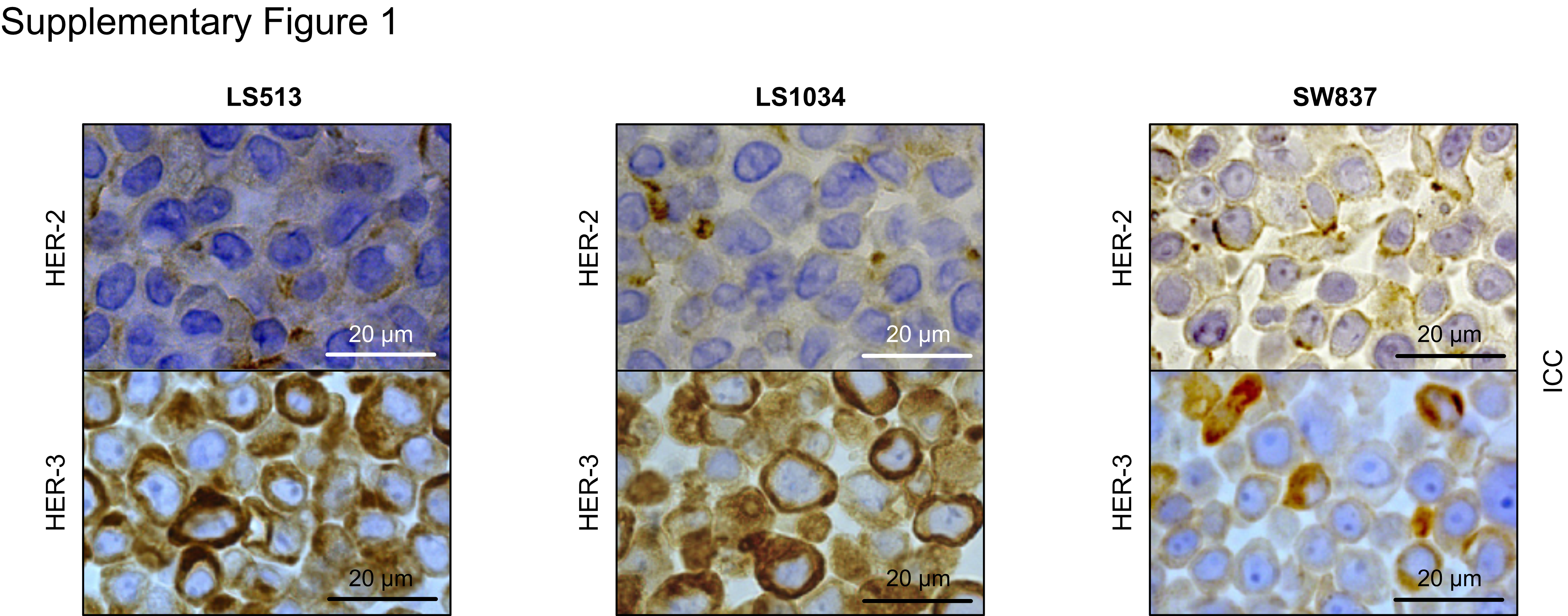

Supplement: Supplementary file 1 — Figure S1. HER-2 and HER-3 status of CRC cell lines. Twelve CRC cell lines were analyzed for membrane expression of HER-2 and HER-3. Overexpression of HER-3 (ICC ≥ 2+) was detected in five cell lines, whereas HER-2 was overexpressed (ICC ≥ 2+) in six cell lines. Depicted are representative HER-2 and HER-3 stainings of LS513, LS1034, and SW837 cells, paraffin embedded. (PNG 5582 kb) [file 12885_2019_6051_MOESM1_ESM.png]

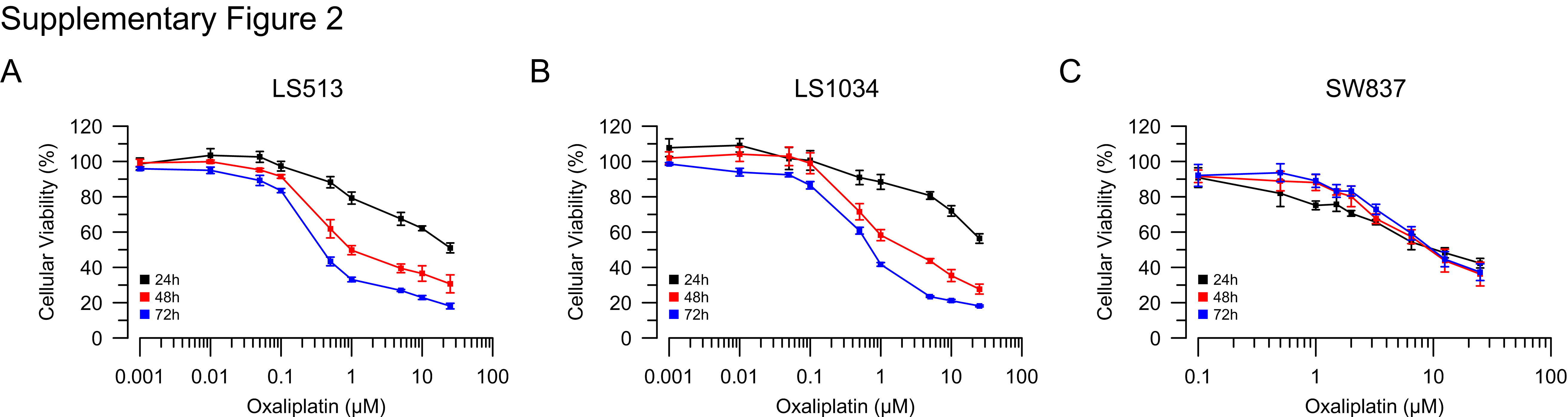

Supplement: Supplementary file 2 — Figure S2. Dose-response curves for oxaliplatin. Cellular viability of LS513 (A), LS1034 (B), and SW837 (C) cells was determined 24 h (black curve), 48 h (red curve), and 72 h (blue curve) after treatment with increasing concentrations of oxaliplatin. All experiments were performed in triplicate, independently repeated three times. (PNG 247 kb) [file 12885_2019_6051_MOESM2_ESM.png]

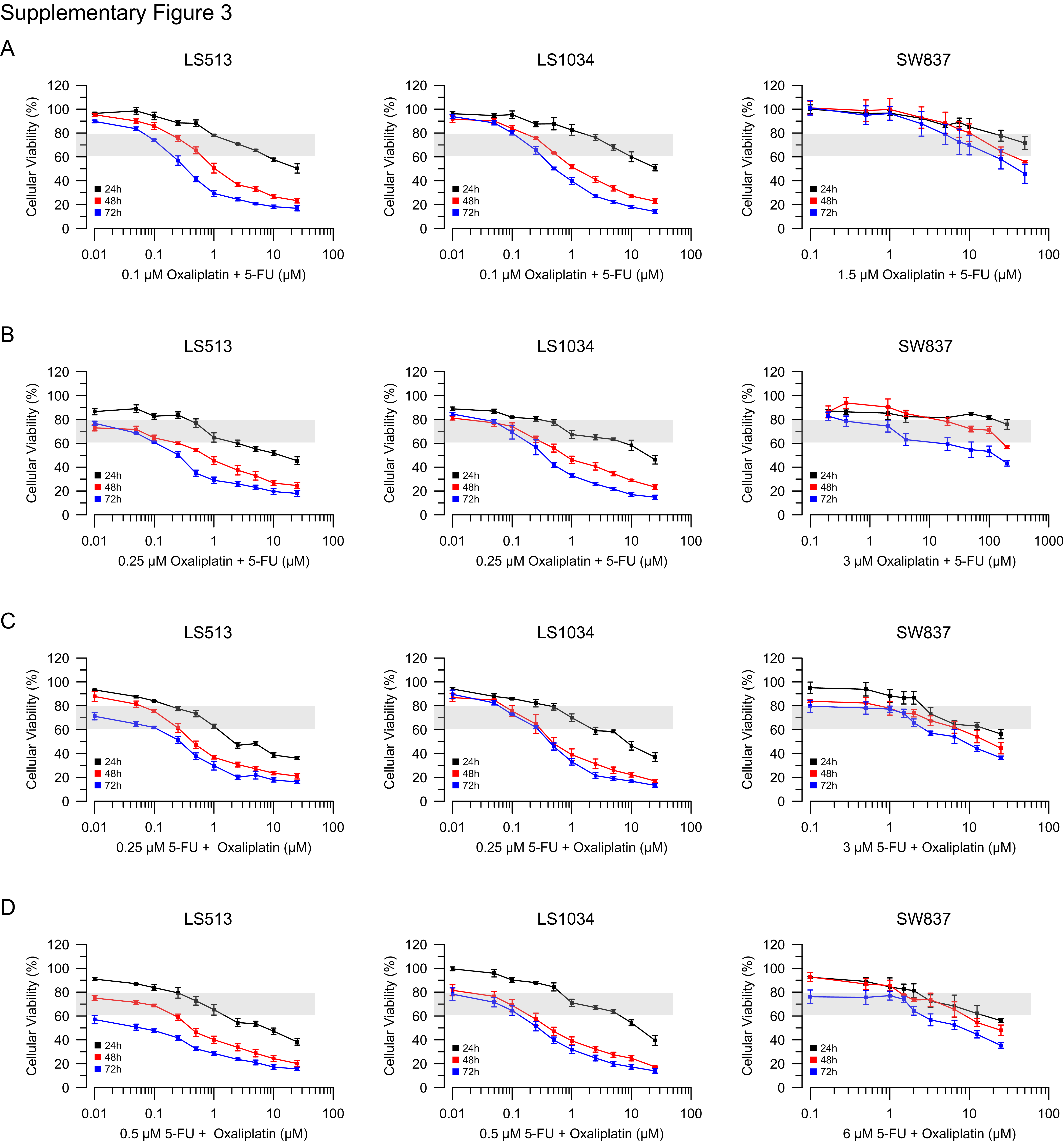

Supplement: Supplementary file 3 — Figure S3. Effect of a combination of 5-FU and oxaliplatin. Cellular viability of LS513, LS1034, and SW837 cells was determined 24 h (black curve), 48 h (red curve), and 72 h (blue curve) after treatment with various concentrations of oxaliplatin and 5-FU. (A + B) Different doses of oxaliplatin were combined with increasing concentrations of 5-FU. (C + D) Different doses of 5-FU were combined with increasing concentrations of oxaliplatin. All experiments were performed in triplicate, independently repeated three times. (PNG 964 kb) [file 12885_2019_6051_MOESM3_ESM.png]

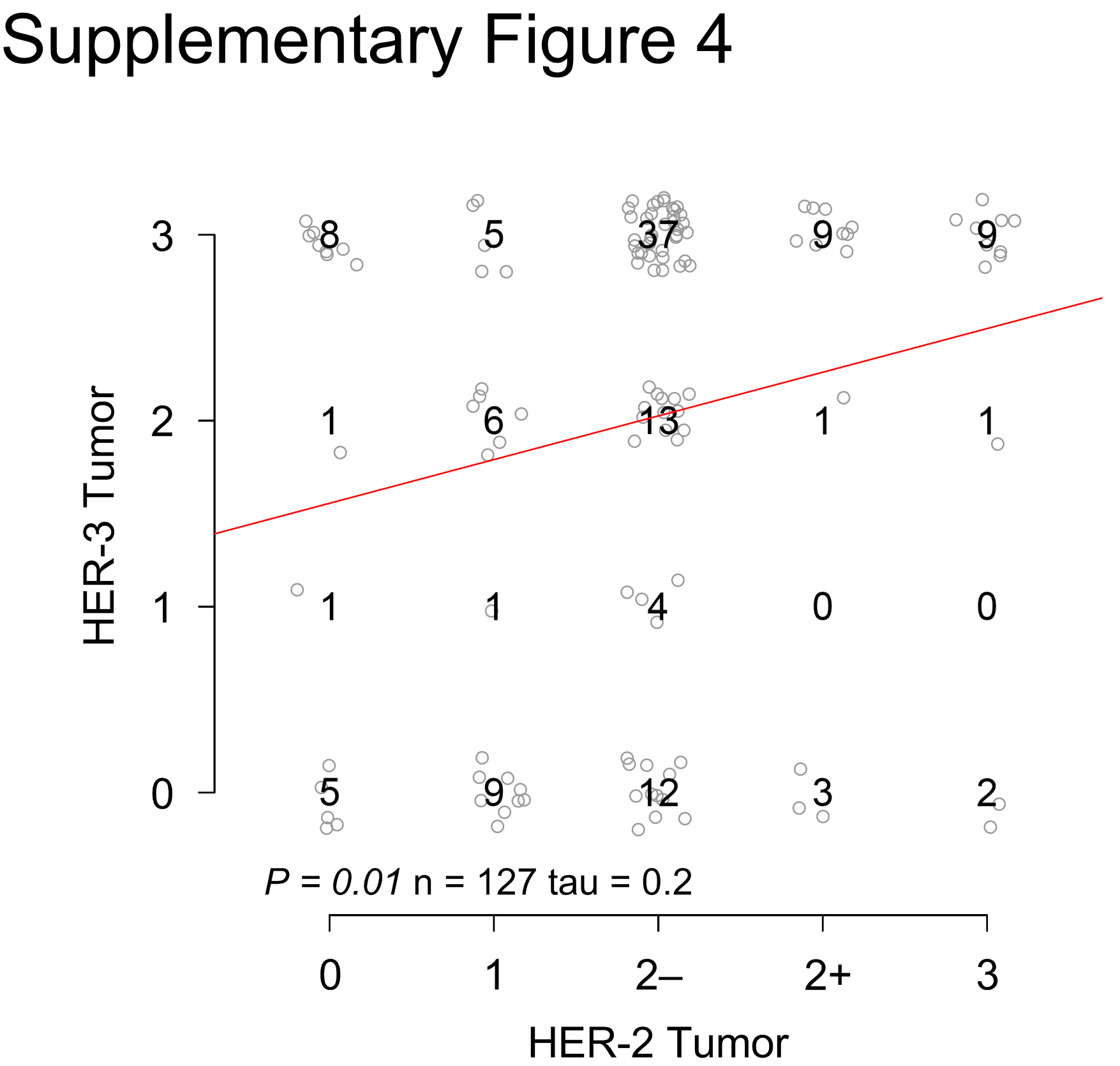

Supplement: Supplementary file 4 — Figure S4. Correlation of HER-2 and HER-3 protein expression in the 127 rectal cancer resection specimens as determined by immunohistochemical staining for HER-2 and HER-3 respectively. Immunohistochemical scoring was performed in 3 different grades (no expression = 0, weak = 1 intermediate = 2 and strong = 3. (PNG 167 kb) [file 12885_2019_6051_MOESM4_ESM.png]
