# Supplementary material for: Combined targeting of HER-2 and HER-3 represents a promising therapeutic strategy in colorectal cancer
Source: BMC Cancer. 2019 Sep 5;19:880. doi: 10.1186/s12885-019-6051-0 (PMC6727342; doi:10.1186/s12885-019-6051-0)
Supplement: Supplementary file 5 — Table S1. EC50s of LS513, LS1034, SW837. (DOC 125 kb) [file 12885_2019_6051_MOESM5_ESM.doc]

Supplementary Table 1. EC50s of LS513, LS1034, SW837

| **Cell Line** | **Drug** | **Duration** | **EC50** |
| --- | --- | --- | --- |
| LS513 | Trastzumab  p-value | 24h  48h  72h  5.2e−18 | 3640000μg/ml  65900000μg/ml  1.08e+09μg/ml  4.1e−10 |
| LS513 | Pertuzumab  p-value | 24h  48h  72h  4.7e−07 | 189000μg/ml  2110000μg/ml  3130000μg/ml  2e−13 |
| LS513 | T-DM1  p-value | 24h  48h  72h  2.1e−09 | 2280μg/ml  72.4μg/ml  25.8μg/ml  4.1e−28 |
| LS513 | Lapatinib  p-value | 24h  48h  72h  0.00065 | 3.96μM  1.68μM  0.686μM  7.2e−32 |
| LS513 | Afatinib  p-value | 24h  48h  72h  1.7e−06 | 6.2μM  1.98μM  0.596μM  1.7e−31 |
| LS513 | Trastzumab + 1µg/ml Pertuzumab  p-value | 24h  48h  72h  0.015 | 462000μg/ml  447000μg/ml  48800μg/ml  1.1e−16 |
| LS513 | Trastuzumab + 20µg/ml Pertuzumab  p-value | 24h  48h  72h  0.49 | 44600μg/ml  2880000μg/ml  7100μg/ml  5.4e−20 |
| LS513 | Pertuzumab + 1µg/ml Trastuzumab  p-value | 24h  48h  72h  1.2e−12 | 28800μg/ml  93100μg/ml  19800μg/ml  1.8e−25 |
| LS513 | Pertuzumab + 20µg/ml Trastuzumab  p-value | 24h  48h  72h  9.9e−10 | 17300μg/ml  456000μg/ml  8420μg/ml  1.1e−19 |
| LS513 | Oxaliplatin  p-value | 24h  48h  72h  2.3e−15 | 48.9μM  2.18μM  0.5μM  2e−37 |
| LS513 | 5-FU + 0.1µM Oxaliplatin  p-value | 24h  48h  72h  1.2e−34 | 22.4μM  1.46μM  0.412μM  6.3e−54 |
| LS513 | 5-FU + 0.25µM Oxaliplatin  p-value | 24h  48h  72h  9.6e−31 | 10.9μM  0.676μM  0.247μM  9.7e−44 |
| LS513 | Oxaliplatin + 0.25µM 5-FU  p-value | 24h  48h  72h  7.8e−26 | 4.1μM  0.711μM  0.193μM  2e−49 |
| LS513 | Oxaliplatin + 0.5µM 5-FU  p-value | 24h  48h  72h  3.8e−42 | 6.48μM  0.521μM  0.0452μM  5.8e−51 |
| LS513 | Trastzumab + 0.25µM 5-FU + 0.1µM Oxaliplatin  p-value | 24h  48h  72h  8.4e−28 | 4650000μg/ml  158000μg/ml  9830000μg/ml  5.1e−13 |
| LS513 | Pertuzumab + 0.25µM 5-FU + 0.1µM Oxaliplatin  p-value | 24h  48h  72h  1.9e−28 | 2660000μg/ml  1.14e+09μg/ml  364000μg/ml  3.3e−07 |
| LS513 | T-DM1 + 0.25µM 5-FU + 0.1µM Oxaliplatin  p-value | 24h  48h  72h  5e−28 | 906μg/ml  27.7μg/ml  11.7μg/ml  3.9e−36 |
| LS513 | Lapatinib + 0.25µM 5-FU + 0.1µM Oxaliplatin  p-value | 24h  48h  72h  1.3e−08 | 10.2μM  2.52μM  2.08μM  2.1e−32 |
| LS513 | Afatinib + 0.25µM 5-FU + 0.1µM Oxaliplatin  p-value | 24h  48h  72h  3.4e−11 | 4.13μM  0.911μM  0.527μM  2.4e−35 |
| LS1034 | Trastzumab  p-value | 24h  48h  72h  0.00011 | 131000μg/ml  5.44e+09μg/ml  2530000μg/ml  7.6e−11 |
| LS1034 | Pertuzumab  p-value | 24h  48h  72h  9.9e−06 | 59900μg/ml  2990000μg/ml  695000μg/ml  2.1e−15 |
| LS1034 | T-DM1  p-value | 24h  48h  72h  1.2e−10 | 1040μg/ml  159μg/ml  61.7μg/ml  1.5e−33 |
| LS1034 | Lapatinib  p-value | 24h  48h  72h  0.00016 | 102μM  5.27μM  4.62μM  6.1e−27 |
| LS1034 | Afatinib  p-value | 24h  48h  72h  0.00061 | 7.36μM  2.32μM  1.59μM  4.7e−34 |
| LS1034 | Trastzumab + 1µg/ml Pertuzumab  p-value | 24h  48h  72h  0.006 | 2190000μg/ml  17600μg/ml  188000μg/ml  8.7e−15 |
| LS1034 | Trastuzumab + 50µg/ml Pertuzumab  p-value | 24h  48h  72h  0.015 | 1.2e+08μg/ml  85800μg/ml  261000μg/ml  8.5e−16 |
| LS1034 | Pertuzumab + 1µg/ml Trastuzumab  p-value | 24h  48h  72h  0.02 | 9850μg/ml  15000μg/ml  2120μg/ml  6.3e−24 |
| LS1034 | Pertuzumab + 100µg/ml Trastuzumab  p-value | 24h  48h  72h  0.18 | 29700µg/ml  96200μg/ml  11900μg/ml  8.9e−16 |
| LS1034 | Oxaliplatin  p-value | 24h  48h  72h  4.5e−13 | 195μM  2.81µM  0.537µM  2.3e−31 |
| LS1034 | 5-FU + 0.1µM Oxaliplatin  p-value | 24h  48h  72h  3.1e−32 | 27μM  1.6μM  0.706μM  1.6e−53 |
| LS1034 | 5-FU + 0.25µM Oxaliplatin  p-value | 24h  48h  72h  1.6e−29 | 17.3µM  1.13µM  0.473μM  1.4e−47 |
| LS1034 | Oxaliplatin + 0.25µM 5-FU  p-value | 24h  48h  72h  2.6e−25 | 7.74μM  0.739μM  0.516μM  7.6e−52 |
| LS1034 | Oxaliplatin + 0.5µM 5-FU  p-value | 24h  48h  72h  1.3e−39 | 11.7μM  0.479μM  0.25μM  2e−53 |
| LS1034 | Trastzumab + 0.25µM 5-FU + 0.25µM Oxaliplatin  p-value | 24h  48h  72h  5e−28 | 672000μg/ml  1590000μg/ml  3.3e+08μg/ml  1.3e−09 |
| LS1034 | Pertuzumab + 0.25µM 5-FU + 0.25µM Oxaliplatin  p-value | 24h  48h  72h  5.5e−36 | 747000μg/ml  4160000μg/ml  190000μg/ml  4.6e−10 |
| LS1034 | T-DM1 + 0.25µM 5-FU + 0.25µM Oxaliplatin  p-value | 24h  48h  72h  3.8e−31 | 1280μg/ml  142μg/ml  23.3μg/ml  8.9e−37 |
| LS1034 | Lapatinib + 0.25µM 5-FU + 0.25µM Oxaliplatin  p-value | 24h  48h  72h  2.9e−10 | 22.6μM  7.39μM  2.08μM  1.3e−24 |
| LS1034 | Afatinib + 0.25µM 5-FU + 0.25µM Oxaliplatin  p-value | 24h  48h  72h  5.1e−09 | 4.24μM  1.1μM  0.537μM  5.2e−31 |
| SW837 | Trastzumab  p-value | 24h  48h  72h  0.0058 | 1.33e+14μg/ml  2.27e+11μg/ml  5.98e+08μg/ml  5e−06 |
| SW837 | Pertuzumab  p-value | 24h  48h  72h  1.5e−10 | 1610000μg/ml  55200μg/ml  28000μg/ml  1.6e−13 |
| SW837 | T-DM1  p-value | 24h  48h  72h  5.6e−08 | 345μg/ml  92.2μg/ml  19.8μg/ml  2.9e−34 |
| SW837 | Lapatinib  p-value | 24h  48h  72h  6.3e−07 | 23.6μM  3.54μM  1.63μM  1.4e−27 |
| SW837 | Afatinib  p-value | 24h  48h  72h  0.00076 | 6.27μM  2.82μM  1.07μM  1.9e−30 |
| SW837 | Trastzumab + 1µg/ml Pertuzumab  p-value | 24h  48h  72h  3e−04 | 66100μg/ml  108000μg/ml  16500μg/ml  1.8e−20 |
| SW837 | Trastuzumab + 100µg/ml Pertuzumab  p-value | 24h  48h  72h  1.3e−05 | 29000μg/ml  293000μg/ml  35500μg/ml  6.3e−22 |
| SW837 | Pertuzumab + 1µg/ml Trastuzumab  p-value | 24h  48h  72h  1.4e−08 | 127000μg/ml  28200000μg/ml  33300μg/ml  6.8e−20 |
| SW837 | Pertuzumab + 20µg/ml Trastuzumab  p-value | 24h  48h  72h  1.1e−09 | 1030000μg/ml  85200μg/ml  17000μg/ml  5.6e−22 |
| SW837 | 5-FU  p-value | 24h  48h  72h  1.2e−13 | 281μM  58.3μM  8.26μM  2.9e−27 |
| SW837 | Oxaliplatin  p-value | 24h  48h  72h  0.1 | 12μM  11.2μM  12.5μM  1.2e−26 |
| SW837 | 5-FU + 1.5µM Oxaliplatin  p-value | 24h  48h  72h  9e−04 | 3030μM  164μM  54.7μM  4.3e−20 |
| SW837 | 5-FU + 3µM Oxaliplatin  p-value | 24h  48h  72h  4.2e−16 | 311000μM  97.1μM  13μM  2.7e−20 |
| SW837 | Oxaliplatin + 3µM 5-FU  p-value | 24h  48h  72h  7.4e−11 | 37.1μM  22.3μM  8.93μM  2.9e−26 |
| SW837 | Oxaliplatin + 6µM 5-FU  p-value | 24h  48h  72h  5.3e−09 | 37.5μM  20.4μM  8.58μM  4.6e−24 |
| SW837 | Trastzumab + 3µM 5-FU + 3µM Oxaliplatin  p-value | 24h  48h  72h  0.043 | 1.52e+09μg/ml  76700μg/ml  1.36e+08μg/ml  8.6e−06 |
| SW837 | Trastzumab + 6µM 5-FU + 3µM Oxaliplatin  p-value | 24h  48h  72h  0.0047 | 5900000μg/ml  45500μg/ml  9.63e+08μg/ml  1.1e−08 |
| SW837 | Pertuzumab + 3µM 5-FU + 3µM Oxaliplatin  p-value | 24h  48h  72h  3.2e−07 | 495000μg/ml  1.27e+13μg/ml  65900000μg/ml  1.3e−05 |
| SW837 | Pertuzumab + 6µM 5-FU + 3µM Oxaliplatin  p-value | 24h  48h  72h  8.8e−06 | 531000μg/ml  1.17e+18μg/ml  17100000μg/ml  0.00028 |
| SW837 | T-DM1 + 3µM 5-FU + 3µM Oxaliplatin  p-value | 24h  48h  72h  2.1e−26 | 3630μg/ml  328μg/ml  13.3μg/ml  1.1e−25 |
| SW837 | T-DM1 + 6µM 5-FU + 3µM Oxaliplatin  p-value | 24h  48h  72h  2.1e−17 | 3550μg/ml  556μg/ml  18.2μg/ml  2.9e−19 |
| SW837 | Lapatinib + 3µM 5-FU + 3µM Oxaliplatin  p-value | 24h  48h  72h  3.3e−05 | 12.2μM  5.13μM  3.47μM  8.6e−28 |
| SW837 | Lapatinib + 6µM 5-FU + 3µM Oxaliplatin  p-value | 24h  48h  72h  0.0034 | 14.8μM  6.29μM  4.33μM  1.2e−24 |
| SW837 | Afatinib + 3µM 5-FU + 3µM Oxaliplatin  p-value | 24h  48h  72h  3e−05 | 9.19μM  3.83μM  2.17μM  1.4e−26 |
| SW837 | Afatinib + 6µM 5-FU + 3µM Oxaliplatin  p-value | 24h  48h  72h  1.3e−05 | 12.9μM  4.41μM  2.07μM  5e−24 |

EC50 = (estimated) effective concentration at 50% remaining viability; 5-FU = 5-Fluorouracil
